# Does supportive supervision intervention improve community health worker knowledge and practices for community management of childhood diarrhea and pneumonia? Lessons for scale-up from Nigraan and Nigraan Plus trials in Pakistan

**DOI:** 10.1186/s12960-021-00641-9

**Published:** 2021-08-17

**Authors:** Wafa Aftab, Suneel Piryani, Fauziah Rabbani

**Affiliations:** Department of Community Health Sciences, The Aga Khan University, Karachi, Stadium Road, P.O Box 3500, Karachi, 74800 Pakistan

**Keywords:** Diarrhea, Feedback, Integrated community case management, Lady health worker, Lady health supervisor, Pneumonia, Supportive supervision, Skills

## Abstract

**Background:**

Lack of programmatic support and supervision is one of the underlying reasons of the poor performance of Pakistan’s Lady Health Worker Program (LHWP). This study describes the findings and potential for scale-up of a supportive supervision intervention in two districts of Pakistan for improving LHWs skills for integrated community case management (iCCM) of childhood diarrhea and pneumonia.

**Methods:**

The intervention comprised an enhanced supervision training to lady health supervisors (LHSs) and written feedback to LHWs by LHSs, implemented in Districts Badin and Mirpur Khas (MPK). Clinical skills of LHWs and LHSs and supervision skills of LHSs were assessed before, during, and after the intervention using structured tools.

**Results:**

LHSs’ practice of providing written feedback improved between pre- and mid-intervention assessments in both trials (0% to 88% in Badin and 25% to 75% in MPK) in the study arm. Similarly, supervisory performance of study arm LHSs was better than that in the comparison arm in reviewing the treatment suggested by workers’ (94% vs 13% in MPK and 94% vs 69% in Badin) during endline skills assessment in both trials. There were improvements in LHWs’ skills for iCCM of childhood diarrhea and pneumonia in both districts. In intervention arm, LHWs’ performance for correctly assessing for dehydration (28% to 92% in Badin and 74% to 96% in MPK), and measuring the respiratory rate correctly (12% to 44% in Badin and 46% to 79% in MPK) improved between baseline and endline assessments in both trials. Furthermore, study arm LHWs performed better than those in comparison arm in classifying diarrhea correctly during post-intervention skills assessment (68% vs 40% in Badin and 96% vs 83% in MPK).

**Conclusion:**

Supportive supervision including written feedback and frequent supervisor contact could improve the performance of community-based workers in managing diarrhea and pneumonia among children. Positive lessons for provincial scale-up can be drawn.

*Trial registration* Both trials are registered with the ‘Australian New Zealand Clinical Trials Registry’. Registration numbers: Nigraan Trial: ACTRN1261300126170; Nigraan Plus: ACTRN12617000309381.

## Background

Under-five mortality remains persistently high in Pakistan at 74/1000 live births and the country has one of the world’s highest neonatal mortality rates (42/1000 live births) in the world [1]. Diarrhea and pneumonia have a high prevalence among Pakistani children—incidence of diarrhea and acute respiratory infections is 19% and 14% according to Pakistan Demographic Health Survey (PDHS) [1]—and the two diseases are responsible for deaths of 90,000 under-five children every year [2]. A large proportion of these deaths is preventable if effective and low-cost interventions such as oral rehydration therapy (ORT), breastfeeding, continued feeding during illness, zinc supplementation, and antibiotics are available and appropriately used [3]. However, only 37% of children under five with diarrhea in Pakistan receive ORS, 8% get zinc supplements and only 46% children with acute respiratory infections or pneumonia receive antibiotics [1]. Improving access and utilization of these lifesaving interventions is crucial if Pakistan is to achieve its sustainable development goal (SDG) 3.2 targets to reduce neonatal mortality to at least 12 per 1000 live births and under-5 mortality to at least 25 per 1000 live births [4, 5].

Access to prompt, appropriate and affordable treatment is critical to reduce childhood mortality due to diarrhea and pneumonia. Community-based interventions involving community health workers (CHWs) have been introduced in many countries to increase the access to services for deprived population for management of these illnesses [6, 7]. World Health Organization (WHO) and UNICEF recommend that integrated community case management (iCCM) of these diseases through CHWs is an effective way of increasing access to quality and affordable services.

CHWs are lay health workers, selected from their communities, and imparted with short-term training [8]. Evidence shows that CHWs can effectively assess, treat, and refer diarrhea and pneumonia cases to health facilities [9]. In Pakistan, National Program for Family Planning and Primary Health Care commonly known as Lady Health Worker’s Program (LHW-P) was launched in 1994 to improve access to community health services in rural and peri-urban areas. Community health workers—called lady health workers (LHWs)—are selected from their local communities based on pre-defined criteria. They are given 15 months of classroom and field-based training. Each LHW is responsible for serving 100–150 households or 1000 population by visiting five to seven households daily. LHWs deliver multiple promotive, preventive and basic curative maternal and child health services including community-based management of diarrhea and pneumonia [10].

An external evaluation of the Program carried out in 2019 found that the program has not been effective in improving health outcomes for infant and child health and that lack of programmatic support and supervision is one of the underlying reasons [11]. An earlier external evaluation reported that 61% of LHWs received no feedback from their supervisors, hampering development of skills in diagnosing and managing ill children in their community [12]. Global evidence also shows that the most common reasons for suboptimal knowledge and skills among CHWs are poor quality training and lack of quality supervision [13]. There is need for more effective and efficient measures of supervision of CHWs for implementation of iCCM. Evidence suggested that supportive supervision improves worker motivation, performance, and quality of care [14–18]. It strengthens the trust relationship between CHWs and supervisors, two-way communication, and teamwork [14, 18]. It promotes problem-solving, cross-learning and skill sharing [18].

In order to address the supervisory gaps in LHWP, trials Nigraan and Nigraan Plus were designed and implemented as sequential clustered randomized controlled trials (RCTs) in Badin and Mirpur Khas (MPK) districts, respectively. Both trials assessed the impact of enhancing structured supportive supervision by LHSs to LHWs’ on CCM of childhood diarrhea and pneumonia knowledge and skills for management of childhood diarrhea and pneumonia in the community. This study compares the findings of the two trials and proposes recommendations for scaling-up this intervention.

## Methods

### Study design and setting

Nigraan and Nigraan Plus trials were cluster randomized control trials conducted in districts Badin (2015) and MPK (2019), in Sindh province.

Badin district is located at a distance of 212 km from the provincial capital, Karachi. It has a population of about 1.35 million, of which 84% is rural, and 24.63% are literate [19, 20]. MPK is an adjoining District 220 km from Karachi, has a total population of 1.5 million, 72% live in a rural setup, and 36.8% are literate [20, 21]. Badin has 11 rural health centers, 34 basic health units and 5 secondary level facilities, 1 district headquarter hospital and 4 taluka headquarter hospitals. A network of about 1100 LHWs, supervised by 36 lady health supervisors (LHSs) cater to maternal and child health needs at community level.

In MPK, there are 6 rural health centers and 38 basic health units, 2 taluka and 1 district headquarter hospital [22]. A total of 983 LHWs, supervised by 39 lady health supervisors (LHSs) provide services in the community [23]. A large number of private healthcare providers deliver care to people in both districts. Incidence of diarrhea and pneumonia in MPK is 30.3% and 10.4% whereas in Badin these are 38.7% and 9.7%, respectively [24].

### Supervision mechanisms in the Lady Health Worker Program

LHWP’s supervision system comprises a network of LHSs each of whom oversees and mentors 25–30 LHWs. An LHS provides hands-on supervision by visiting each LHW reporting to her at least twice every month.

During these visits, the LHS accompanies the LHW during household visits to the community and provides verbal feedback to the LHW on her performance. All LHWs provide data from the patients they attended during the month to LHSs in monthly meetings; LHSs collate this data and report it to the district program office every month.

### Sampling and data collection

#### Sample size

Based on incidence of pneumonia in children < 5, in Badin, the sample size was estimated at 34 LHSs to achieve 80% power for estimating the effect of the intervention at 95% confidence interval. Thirty-four functional LHSs were allocated randomly to either intervention or control arm with a ratio of 1:1. Of 25–30 LHWs working under each LHS, five were randomly recruited, to a total of 85 LHWs in each arm. Similarly using an assumption of 15% increase in ORS and antibiotic use, estimated sample size was 16 LHSs in each arm in MPK. Thirty-two functional LHSs were randomized: 16 each in intervention and control groups. Of 25–30 LHWs working under each LHS, five were randomly recruited, to a total of 80 LHWs in each group.

### Intervention design

The intervention in the trials consisted of an enhanced supervision training for the LHSs in the intervention arm. After the training the LHSs regularly visited LHWs in the intervention arm in the field and provided community-based enhanced supervision through written feedback. To facilitate communication and to allow surveillance and reporting of diarrhea and pneumonia cases, all LHSs were given mobile phones and given a monthly communication allowance so that they could communicate with LHWs.

#### Development of enhanced supervisory training curriculum and LHS training in both districts

A detailed training manual was developed which provides training objectives, pedagogy and training content for the enhanced supervision intervention. Multiple instructional methodologies were used including face-to-face interactive lectures supplemented with role-play, audio-visual aids, simulated exercises, and hands-on training in a health facility.

Since workers in the LHWP had not had an iCCM training in about a decade, a 2-day refresher training based on existing LHWP guidelines was provided to all study LHSs (intervention and control).

LHSs in the intervention arm were then provided an additional 4-day training based on the enhanced supervisory training curriculum to improve their clinical and supervisory skills, including skills on effectively providing feedback.

#### Improved communication and case surveillance

A short message service (SMS) communication system was added to the existing management information system of LHWP to track diarrhea and pneumonia cases in both trials. LHWs identified cases in the community and informed LHSs after which LHS conducted a supervisory visit within 24 h. During the visit, LHS reviewed the LHWs case management practices and provided written feedback. The SMS surveillance system tracked cases reported by LHWs to LHSs. A log was maintained of all reported cases and from this list, some cases were randomly selected for skill assessment of LHWs and LHSs periodically.

#### Enhanced supportive supervision through written feedback cards

Besides supervision skills training for LHSs, use of modified or additional supervision tools, provision of written feedback to LHWs, active and prompt case reporting by LHWs were part of the intervention package [25]. Intervention arm LHSs provided written feedback to LHWs using a structured written feedback card. This activity added approximately 10 min to each visit’s duration. LHSs in both arms provided verbal feedback as part of the existing program protocol.

#### LHS and LHW skill assessments

Diarrhea and pneumonia management skills of all study LHWs and LHSs were studied periodically. In MPK, the skills of all LHWs were assessed during midline and endline assessments, while for the baseline assessment, 51 LHWs (27 intervention and 24 control) were selected randomly for assessment of skills related to case management of diarrhea and 58 LHWs (28 Intervention and 30 Control) for assessment of skills pertaining to pneumonia. Similarly, in Badin 25 LHWs were chosen randomly for assessing CCM skills for diarrhea and pneumonia at baseline midline and post-intervention.

#### Skill assessment scorecards

In both trials, clinical and supervision skills were assessed by independent evaluators using structured clinical and supervision skill assessments scorecards. The iCCM clinical skill assessment scorecard was developed based on LHWP curriculum. This tool was used to assess history taking, physical examination, diagnosis, treatment and counselling skills of LHWs and LHSs. A structured scorecard was used to assess LHSs’ supervision and mentoring skills. The clinical mentoring section assessed LHSs’ ability to evaluate LHWs’ clinical skills whereas the supervision component assessed LHSs’ provision of verbal and written feedback and her demeanor.

### Data analysis

For both trials data were analyzed using SPSS version 21 [26]. Descriptive statistics were computed for socio-demographic characteristics and skill levels of LHWs/LHSs. Mann–Whitney *U*, independent-sample *t*, Pearson’s Chi-square tests were used to assess socio-demographic difference between intervention and comparison arm. A *p*-value < 0.05 was considered to be significant.

### Ethical consideration

Ethical approval for both trials was obtained from the Aga Khan University Ethical Review Committee. An informed written consent was obtained from each LHS and LHW for their involvement at the beginning of each trial. Both trials are registered with the ‘Australian New Zealand Clinical Trials Registry’. Registration numbers: Nigraan Trial: ACTRN1261300126170; Nigraan Plus: ACTRN12617000309381.

## Results

### Socio-demographic characteristics

In MPK, the intervention arm LHSs and LHWs’ median age, education, median years of experience with LHWP, lives in house with under 5 years child, and median time to reach health facility were comparable with comparison arm LHSs and LHWs, respectively. However, compared to study arm LHWs, comparison arm LHWs take more time to reach health facility. Similarly, in Badin, LHSs and LHWs in both arms were comparable in most socio-demographic characteristics except for a higher median age in comparison arm LHWs and LHSs, and higher median years of experience in comparison arm LHSs (Table 1).

**Table 1:** Socio-demographic characteristics

Characteristic	Nigraan (Badin)				Nigraan Plus (MPK)			
	LHSs (*n* = 34)		LHWs (*n* = 168)		LHSs (*n* = 32)		LHWs (*n* = 160)	
	Intervention (*n* = 17)	Control (*n* = 17)	Intervention (*n* = 84)	Control (*n* = 84)	Intervention (*n* = 16)	Control (*n* = 16)	Intervention (*n* = 80)	Control (*n* = 80)
Median age (min, max)*^†^	37 (29, 45)	40 (32, 48) ‡	33 (22, 55)	36.5 (24, 59)^‡^	45 (40, 67)	43 (38, 57)	39 (25, 57)	41 (26, 59)
Education *n* (%)***^†^								
Secondary	5 (29.4)	3 (17.6)	74 (89.2)	79 (94.0)	3 (20.0)	4 (28.6)	65 (92.9)	66 (90.4)
Tertiary	12 (70.6)	14 (82.4)	9 (10.8)	5 (6.0)	12 (80)	14 (71.4)	5 (7.1)	7 (9.6)
Had previous experience before joining LHWP *n* (%)**^†^	5 (29.4)	7 (41.2)	7 (8.3)	4 (4.8)	7 (58.3)^‡^	1 (12.5)	6 (11.3)	2 (6.7)
Median years of experience with LHWP (min, max)*^†^	12 (0, 20)	14 (8, 20)^‡^	12 (0, 20)	13 (0, 21)	22 (14, 25)	20.5 (14, 24)	18 (1, 26)	17 (2, 25)
Marital status *n* (%)***^†^								
Married	17 (100)	17 (100)	67 (79.8)	73 (86.9)	14 (93.3)	9 (81.8)	64 (88.9)	59 (90.8)
Single			14 (16.7)	8 (9.5)	1 (6.7)	1 (9.1)	7 (9.7)	5 (7.7)
Divorced			1 (1.2)	2 (2.4)	0 (0)	1 (9.1)	0 (0)	1 (1.5)
Widowed			2 (2.4)	1 (1.2)	0 (0)	0 (0)	1 (1.4)	0 (0)
Lives in a household with < 5-year child *n* (%)***^†^	9 (52.9)	6 (35.3)	39 (46.4)	45 (53.6)	2 (14.3)	5 (38.5)	26 (38.8)	30 (40.0)
Median time to reach health facility (min, max)*^†^	15 (0,60)	20 (10, 60)	15 (0, 60)	20 (3, 60)	22 (14, 25)	20.5 (14, 24)	20 (5, 60)	25 (5, 120)^‡^

*LHWP* Lady Health Worker Program, *LHWs* lady health workers, *LHSs* lady health supervisors

Statistical tests applied for significance testing: *Mann–Whitney *U*-test. **Independent sample *t*-test. ***Pearson’s Chi-square

^†^Some missing values for LHWs in both trials and LHSs in MPK

^‡^*p*-value > 0.05

### Lady health worker performance for iCCM of diarrhea

Improvement was observed in intervention the arm LHWs’ performance for iCCM of diarrhea between first (pre-intervention) and third assessments in both trials. For instance, enquiring about danger signs (60% to 96% in Badin and 89% to 100% in MPK), correctly assessing for dehydration (28% to 92% in Badin and 74% to 95% in MPK), correctly classifying the illness (24% to 68% in Badin and 85% to 96% in MPK), advising correct treatment (12% to 28% in Badin and 63% to 84% in MPK). Comparison arm showed little improvement for most of the indicators in Badin and few indicators in MPK. In comparison arm, the proportion of LHWs correctly assessing for dehydration increased in Badin trial (28% to 64%), but decreased in MPK (71% to 26%). Similarly, comparison arm LHWs’ performance for counselling the caregivers to seek care immediately if child has danger signs of diarrhea improved in Badin (40% to 96%) while it deteriorated in MPK (63% to 19%). Furthermore, comparison arm LHWs performance indicators such as classifying diarrhea correctly (28% to 40% in Badin and 75% to 83%) and referring the child to doctor for correct indication (36% to 92% in Badin and 54% to 66% in MPK) improved in both trials (Table 2).

**Table 2:** LHWs’ skills for diarrhea

Skills	Nigraan (Badin)						Nigraan Plus (MPK)					
	Performed correctly Pre-intervention		Performed correctly Mid-intervention		Performed correctly Post-intervention		Performed correctly Pre-intervention		Performed correctly Mid-intervention		Performed correctly Post-intervention	
	Intervention *n* = 25 No. (%)	Control *n* = 25 No. (%)	Intervention *n* = 25 No. (%)	Control *n* = 25 No. (%)	Intervention *n* = 25 No. (%)	Control *n* = 25 No. (%)	Intervention *n* = 27 No. (%)	Control *n* = 24 No. (%)	Intervention *n* = 80 No. (%)	Control *n* = 79^a^ No. (%)	Intervention *n* = 80 No. (%)	Control *n* = 80 No. (%)
Danger signs												
Enquires about danger signs	15 (60)	13 (52)	16 (64)	12 (48)	24 (96)	23 (92)	24 (89)	20 (83)	80 (100)	64 (80)	80 (100)	70 (88)
Diagnosis and management												
Correctly assesses for dehydration	7 (28)	12 (48)	18 (72)	12 (48)	23 (92)	16 (64)	20 (74)	17 (71)	76 (95)	42 (53)	76 (95)	21 (26)
Classifies the illness correctly	6 (24)	7 (28)	18 (72)	11 (44)	17 (68)	10 (40)	23 (85)	18 (75)	71 (89)	62 (79)	77 (96)	66 (83)
Advises the correct treatment	3 (12)	6 (24)	10 (40)	5 (20)	7 (28)	7 (28)	17 (63)	20 (83)	31 (39)	40 (51)	67 (84)	34 (43)
Refers for the correct indication	14 (56)	9 (36)	21 (84)	21 (84)	24 (96)	23 (92)	10 (37)	23 (54)	70 (87)	29 (37)	72 (90)	53 (66)
Counselling												
Tells the caregiver to continue feeding the child	10 (40)	10 (40)	21 (84)	11 (44)	25 (100)	25 (100)	26 (96)	23 (96)	79 (99)	58 (73)	80 (100)	71 (89)
Tells caregiver to seek care immediately if the child has dehydration, blood in stools, fever, or fits	14 (56)	10 (40)	21 (84)	19 (76)	25 (100)	24 (96)	22 (82)	15 (63)	70 (88)	8 (10)	71 (89)	15 (19)

^a^Skills of one LHW could not be assessed in midline due to personal reasons

### Lady health worker performance for iCCM of pneumonia

Improvement in intervention arm LHW skills for iCCM of pneumonia was observed for some indicators between pre-intervention and post-intervention assessments in Badin and MPK. LHWs performance for measuring the respiratory rate correctly increased considerably between first and third assessments in intervention arm of both trials (12% to 44% in Badin and 46% to 76% in MPK). Similarly, the proportion of LHWs counselling caregivers to seek care immediately if child has danger signs increased greatly between first and third assessment in intervention arms of Badin and MPK (36% to 100% in Badin and 50% to 83%). Likewise, other indicators of performance such as classifying illness correctly and advising correct treatment also improved in intervention arm of both trials (Table 3).

**Table 3:** LHWs’ skills for pneumonia

Skills	Nigraan (Badin)						Nigraan Plus (MPK)					
	Performed correctly Pre-intervention		Performed correctly Mid-intervention		Performed correctly Post-intervention		Performed correctly Pre-intervention		Performed correctly Mid-intervention		Performed correctly Post-intervention	
	Intervention *n* = 25 No. (%)	Control *n* = 25 No. (%)	Intervention *n* = 25 No. (%)	Control *n* = 25 No. (%)	Intervention *n* = 25 No. (%)	Control *n* = 25 No. (%)	Intervention *n* = 28 No. (%)	Control *n* = 30 No. (%)	Intervention *n* = 80 No. (%)	Control *n* = 79^a^ No. (%)	Intervention *n* = 80 No. (%)	Control *n* = 80 No. (%)
Danger signs												
Enquires about danger signs	4 (16)	14 (56)	16 (64)	9 (36)	23 (92)	24 (96)	21 (75)	26 (87)	79 (99)	58 (73)	78 (97.5)	54 (68)
Diagnosis and management												
Assesses for respiratory rate correctly	3 (12)	1 (4)	13 (52)	5 (20)	11 (44)	12 (48)	13 (46)	14 (47)	61 (76)	14 (18)	61 (76)	7 (9)
Classifies the illness correctly	2 (8)	5 (20)	16 (64)	09 (36)	11 (44)	10 (40)	25 (89)	28 (93)	73 (91)	53 (67)	73 (91)	65 (81)
Advises the correct treatment	0 (0)	0 (0)	2 (8)	0 (0)	6 (24)	6 (24)	17 (61)	15 (50)	24 (30)	(19)	56 (70)	15 (19)
Refers for correct indication	9 (36)	13 (52)	25 (100)	19 (76)	24 (96)	25 (100)	15 (54)	10 (33)	66 (83)	25 (32)	65 (81)	32 (40)
Counselling												
Tells the caregiver to continue feeding the child	2 (8)	7 (28)	20 (80)	16 (64)	25 (100)	25 (100)	24 (86)	24 (80)	79 (99)	58 (73)	78 (98)	64 (80)
Tells caregiver to seek care immediately if child has fever, becomes drowsy/unconscious or has seizures	9 (36)	11 (44)	21 (84)	19 (76)	25 (100)	23 (92)	14 (50)	13 (43)	66 (83)	11 (14)	66 (83)	17 (21)

^a^Skills of one LHW could not be assessed in midline due to personal reasons

### Lady health supervisor performance for iCCM of pneumonia

The proportion of LHSs correctly counting the respiratory rate increased in intervention arm of both trials between first and third surveys (18% [*n* = 16] to 80% [*n* = 15] in Badin and 42% [*n* = 12] to 88% [*n* = 16] in MPK). Similarly, intervention arm LHSs’ performance of correctly classifying illness enhanced between pre- and post-intervention assessments in Badin trial (0% to 100%), but remained unchanged in MPK (was already 83% at baseline).

### Lady health supervisor performance for iCCM of diarrhea

Comparing baseline to endline survey, LHSs’ practice of assessing the dehydration correctly improved in intervention arm of both trials (6% to 100% in Badin and 92% to 100% in MPK). Likewise, LHSs’ skill of classifying illness correctly was relatively high in intervention arm of Badin (62% to 73%) while exhibiting a slight decline in MPK (92% to 88%) between pre- and post-intervention assessments.

### Lady health supervisors’ supervisory performance

In Badin and MPK trials, most of the indicators of intervention arm LHSs’ supervisory performance showed improvement between pre- and post-intervention assessments. In both trials, LHSs practice of giving written feedback improved between baseline and second assessments (0 to 88% in Badin and 25% to 75% in MPK). Furthermore, in intervention arm, the practice of LHSs giving verbal feedback improved between pre- and post-intervention assessments in both Badin and MPK trials. In terms of clinical supervision, the proportion of LHS reviewing treatment suggested by LHWs increased between first and third assessments in both trials (50% to 94% in Badin and 38% to 94% in MPK) (Table 4).

**Table 4:** Supervisory performance of lady health supervisors

Skills	Nigraan (Badin)						Nigraan Plus (MPK)					
	Performed correctly Pre-intervention		Performed correctly Mid-intervention		Performed correctly Post-intervention		Performed correctly Pre-intervention		Performed correctly Mid-intervention		Performed correctly Post-intervention	
	Intervention *n* = 12 No. (%)	Control *n* = 11 No. (%)	Intervention *n* = 16 No. (%)	Control *n* = 16 No. (%)	Intervention *n* = 16 No. (%)	Control *n* = 16 No. (%)	Intervention *n* = 16 No. (%)	Control *n* = 16 No. (%)	Intervention *n* = 16 No. (%)	Control *n* = 16 No. (%)	Intervention *n* = 16 No. (%)	Control *n* = 16 No. (%)
Clinical mentorship												
Asks LHW to describe case history and findings	12 (100)	6 (54)	16 (100)	13 (81)	14 (88)	13 (81)	15 (94)	15 (94)	15 (94)	9 (56)	15 (94)	13 (81)
Probes for inconsistencies and missing information	5 (42)	2 (18)	14 (88)	10 (63)	12 (75)	9 (56)	11 (69)	11 (69)	15 (94)	6 (38)	15 (94)	11 (69)
Reviews diagnosis with LHW and correlates it with the child findings	3 (25)	1 (9.1)	13 (81)	6 (38)	11 (69)	5 (31)	13 (81)	13 (81)	15 (94)	7 (44)	15 (94)	7 (44)
Demonstrates correct method for clinical examination to the LHW	1 (8)	0 (0)	12 (75)	3 (19)	12 (75)	9 (56)	11 (69)	11 (69)	10 (63)	6 (38)	10 (62)	2 (13)
Reviews treatment suggested by LHW	6 (50)	2 (18)	5 (31)	2 (13)	15 (94)	11 (69)	6 (38)	9 (56)	15 (94)	12 (75)	15 (94)	2 (13)
Discusses indication present in the sick child for suggested management with LHW	7 (58)	3 (27)	11 (69)	4 (25)	13 (81)	10 (63)	11 (69)	9 (56)	14 (88)	7 (44)	14 (88)	3 (19)
Supervisory performance												
Explains the purpose of the supervisory visit to the LHW and clearly spells out her expectations from LHW	8 (67)	5 (46)	14 (88)	11 (69)	12 (75)	7 (44)	13 (81)	11 (69)	16 (100)	9 (56)	16 (100)	6 (38)
Provides LHW with verbal feedback of performance for given child	3 (25)	0 (0)	14 (88)	11 (69)	14 (88)	14 (88)	13 (81)	12 (75)	16 (100)	7 (44)	16 (100)	9 (56)
Provides written performance feedback to the LHW during the visit^a^	0 (0)	(0)	14 (88)	1 (6)	8 (50)	0 (0)	4 (25)	5 (31)	12 (75)	0 (0)	Not applicable	Not applicable 0
Demonstrates encouraging and appreciative attitude towards the LHW	3 (25)	0 (0)	14 (88)	6 (38)	13 (81)	13 (81)	4 (25)	3 (19)	12 (75)	2 (13)	12 (75)	6 (38)

^a^Written feedback card distribution was not assessed post-intervention as they were not available

## Discussion

The results of Badin and MPK trials depict that overtime in the study arm there was improvement in LHSs and LHW performance for correctly assessing dehydration, counting respiratory rate, classifying the illness, advising correct treatment, counselling caregivers to continue feeding and seek care immediately if child has danger signs of diarrhea or pneumonia. There was also improvement in clinical mentoring and supervision by the LHSs. There was high uptake of feedback provision by LHSs and this was largely maintained throughout the study especially for the verbal component. Moreover, LHSs practice of providing written feedback improved as a result of the supportive supervision intervention.

These findings are similar to results of a study conducted in Kenya, which showed that the training along with clinical coaching and continuous supportive supervision improved and maintained CHWs’ skills related to iCCM of diarrhea and pneumonia [27]. Additionally, a study from South Sudan showed an improvement and retention of correct disease identification and treatment skills of CHWs following a supportive supervision intervention [28].

Skills-based training loses its impact if regular supervision and support are not provided [29, 30]. A study conducted in Guinea-Bissau reported that CHWs’ skills of diagnosis and management of childhood diarrhea improved following training but 3 months later the effectiveness of training decreased [29]. This can also be reflected from the results of both trials, poor skills at baseline and subsequent improvements in skills of LHSs and LHWs. This implies that CHWs can improve skills mastery and retention following uninterrupted supportive supervision and monitoring.

Frequency of providing feedback during the supervisory visits improved in both trials. Feedback is an essential component of supportive supervision, and it influences the motivation and performance of workers. A study conducted in Uganda showed that CHWs’ performance in managing diarrhea, pneumonia, and malaria increased five times when workers received feedback from their supervisor [31].

Some improvements were also noticed in comparison arm of both trials. This is not surprising. Refresher training was provided to comparison arm which could have brought improvements in the outcome indicators. Additionally, monitoring of comparison arm LHWs was also carried out, which could have motivated comparison arm LHSs and LHWs to update their knowledge through available local resources. Furthermore, all LHSs of the district (both from study and comparison arm) assemble every month at the same office for a collective appraisal meeting as per LHWPs administrative setup. Moreover, it is much harder to limit the spread of intervention if the participants of intervention and comparison are from the same geographical locality [32].

The improvements in the intervention arm could have been more substantial. However, a number of contextual factors could have hindered the study implementation or intervention effectiveness such as lack of transportation facilities, inadequate supply of medicines, excessive burden of ancillary activities on LHWs and LHSs, and delays in salary disbursement [11]. Lack of transportation could also have prevented LHSs from making frequent supervision visits.

Our study has some potential limitations. Both intervention and control arms were located in the same districts so there may have been some transfer of knowledge between intervention and control LHWs and LHSs. This may have led to some underestimation of the improvement in the intervention arm. Due to the Hawthorne effect, health workers in both intervention and control arms may have performed in a different manner than they normally would leading to some differences in our assessment and real-world behavior.

The findings of both trials have essential implications for scaling-up the intervention. Since 2010 provincial governments have been given more responsibility for gradually taking over the funding and managing of the LHW programs. Their plans for reforms and upscaling of the program can benefit from these findings. Recent LHWP evaluations also suggest that improvement is needed [11]. Findings of both trials therefore suggest that enhancing the skills of LHSs to provide frequent, quality supportive supervision to LHWs can be one way of improving performance of the community-based services of LHWs for childhood illnesses. Future research could focus on effective approaches to sustainably embed these interventions in existing health systems and assess effectiveness in terms of population level impact such as on morbidity and mortality indicators.

## Conclusion

The study found improvements in LHSs and LHWs performance for correctly assessing dehydration, measuring respiratory rate, classifying the illness, advising correct treatment, counselling caregivers to continue feeding and seek care immediately if child has danger signs of diarrhea or pneumonia. There was also progress in LHSs clinical mentoring and supervision. The findings of this study advocate potential benefits of implementing a supportive supervision intervention through LHSs with the potential to improve iCCM of childhood diarrhea and pneumonia. This can serve as a reference for scaling-up the intervention at provincial level. Training supervisors to provide detailed and frequent feedback in a supportive manner can improve the quality of care provided by the LHWs in large scale programs. Planners of programs at scale must consider the budgetary and operational aspects of such investment in human resources for sustainable delivery of quality care at community level.

## Data Availability

Data can be made available on request.

## Declarations

### Dissemination declaration

Dissemination of results to study participants or patient organizations is not applicable for this study.

### Public and patient involvement statement

Neither patients nor the public were involved in the conduct of this study.

### Transparency statement

As guarantor, the senior author affirms that the manuscript is an honest, accurate, and transparent account of the study being reported; that no important aspects of the study have been omitted; and that any discrepancies from the study as originally planned have been explained.

## References

[1] National Institute of Population Studies (NIPS) [Pakistan] and ICF (2018). Pakistan demographic and health survey 2017–18.

[2] WHO. Global health observatory data repository. Causes of child deaths. Number of deaths by causes. 2018. https://apps.who.int/gho/data/view.main.ghe1002015-CH3?lang=en.

[3] Bhutta ZA, Das JK, Walker N, Rizvi A, Campbell H, Rudan I (2013). Interventions to address deaths from childhood pneumonia and diarrhoea equitably: what works and at what cost?. Lancet.

[4] UNICEF (2016). One is too many: ending child deaths from pneumonia and diarrhoea.

[5] Ministry of Planning Development. Sustainable development goals. 2018. https://www.sdgpakistan.pk/web/goals/goal3.

[6] Winch PJ, Gilroy KE, Wolfheim C, Starbuck ES, Young MW, Walker LD (2005). Intervention models for the management of children with signs of pneumonia or malaria by community health workers. Health Policy Plan.

[7] Gilroy K, Winch P. Management of sick children by community health workers. Intervention models and programme examples. 2006.

[8] Lehmann U, Sanders D (2007). Community health workers: what do we know about them.

[9] Young M, Wolfheim C, Marsh DR, Hammamy D (2012). World Health Organization/United Nations Children’s Fund joint statement on integrated community case management: an equity-focused strategy to improve access to essential treatment services for children. Am J Trop Med Hyg.

[10] Hafeez A, Mohamud BK, Shiekh MR, Shah SAI, Jooma R (2011). Lady health workers programme in Pakistan: challenges, achievements and the way forward. JPMA J Pak Med Assoc.

[11] Oxford Policy Management. Lady Health Worker Programme, Pakistan. Performance evaluation. Evaluation Report 2019. United Kingdom; 2019. t.ly/u5qn.

[12] Oxford Policy Management (2009). Lady health worker programme: external evaluation of the national programme for family planning and primary health care. Quantitative survey report.

[13] Glenton C, Colvin CJ, Carlsen B, Swartz A, Lewin S, Noyes J, et al. Barriers and facilitators to the implementation of lay health worker programmes to improve access to maternal and child health: qualitative evidence synthesis. Cochrane Database Syst Rev. 2013;(10). 10.1002/14651858.CD010414.pub2 PMC6396344 24101553

[14] Bailey C, Blake C, Schriver M, Cubaka VK, Thomas T, Martin HA (2016). A systematic review of supportive supervision as a strategy to improve primary healthcare services in Sub-Saharan Africa. Int J Gynecol Obstet.

[15] Marquez L, Kean L. Making supervision supportive and sustainable: new approaches to old problems. MAQ Paper No. 4. Supplement to population reports, Vol XXX, Issue 4. 2011.

[16] Bradley S, Kamwendo F, Masanja H, de Pinho H, Waxman R, Boostrom C (2013). District health managers’ perceptions of supervision in Malawi and Tanzania. Hum Resour Health.

[17] Hill Z, Dumbaugh M, Benton L, Kaellander K, Strachan D, Ten Asbroek A (2014). Supervising community health workers in low-income countries—a review of impact and implementation issues. Glob Health Action.

[18] Kok MC, Vallières F, Tulloch O, Kumar MB, Kea AZ, Karuga R (2018). Does supportive supervision enhance community health worker motivation? A mixed-methods study in four African countries. Health Policy Plan.

[19] Pakistan Bureau of Statistics. Badin District at a glance. Pakistan Bureau of Statistics. 2013. http://www.pbs.gov.pk/content/district-glance-badin. Accessed 28 Apr 2020.

[20] Pakistan Bureau of Statistics. Block wise provisional summary results of 6th population & housing census-2017. Pakistan Bureau of Statistics. 2018. http://www.pbs.gov.pk/content/block-wise-provisional-summary-results-6th-population-housing-census-2017-january-03-2018. Accessed 16 Apr 2019.

[21] Pakistan Bureau of Statistics. Mirpur Khas district at a glance. 2013. http://www.pbs.gov.pk/content/district-glance-mirpur-khas.

[22] Bureau of Statistics, Government of Sindh. Health Profile of Sindh, 2016. Karachi: Bureau of Statistics, Government of Sindh; 2016. http://sindhbos.gov.pk/wp-content/uploads/2017/01/Health-Profile-of-Sindh-by-District-2016.pdf.

[23] UNDP Pakistan & Government of Sindh (2012). Report on The status of millennium development goals sindh.

[24] Sindh Bureau of Statistics, UNICEF (2015). Sindh multiple indicator cluster survey 2014, final report.

[25] Aftab W, Rabbani F, Sangrasi K, Perveen S, Zahidie A, Qazi SA (2018). Improving community health worker performance through supportive supervision: a randomised controlled implementation trial in Pakistan. Acta Paediatr.

[26] Corp IBM (2012). IBM SPSS statistics for Macintosh.

[27] Shiroya-Wandabwa M, Kabue M, Kasungami D, Wambua J, Otieno D, Waka C (2018). Coaching community health volunteers in integrated community case management improves the care of sick children under-5: experience from Bondo, Kenya. Int J Integr Care.

[28] Rosales A, Hedrick J, Amet KK, Walumbe E, Otieno J, Achan R (2014). Maintaining knowledge and technical skills among illiterate frontline community health workers (HHPs) delivering integrated Community Case Management (iCCM) in South Sudan.

[29] Lopes SC, Cabral AJ, de Sousa B (2014). Community health workers: to train or to restrain? A longitudinal survey to assess the impact of training community health workers in the Bolama Region, Guinea-Bissau. Hum Resour Health.

[30] Rowe SY, Olewe MA, Kleinbaum DG, McGowan JE, McFarland DA, Rochat R (2007). Longitudinal analysis of community health workers’ adherence to treatment guidelines, Siaya, Kenya, 1997–2002. Trop Med Int Health.

[31] Bagonza J, Kibira SP, Rutebemberwa E (2014). Performance of community health workers managing malaria, pneumonia and diarrhoea under the community case management programme in central Uganda: a cross sectional study. Malar J.

[32] English M, Schellenberg J, Todd J (2011). Assessing health system interventions: key points when considering the value of randomization. Bull World Health Organ.

